# Effect of Torsion Stress on the Offset and Sensitivity of Diagonal and Off-Diagonal GMI in Amorphous Wires †

**DOI:** 10.3390/s18124121

**Published:** 2018-11-24

**Authors:** Julie Nabias, Aktham Asfour, Jean-Paul Yonnet

**Affiliations:** Univ. Grenoble Alpes, CNRS, Grenoble INP, G2ELab, 38000 Grenoble, France; chickichik@gmail.com (J.N.); Jean-Paul.Yonnet@g2elab.grenoble-inp.fr (J.-P.Y.)

**Keywords:** GMI sensor, diagonal, off-diagonal, sensitivity, offset, torsion stress, parameter of influence

## Abstract

In this paper, the torsional stress effect on Giant Magneto-Impedance (GMI) was studied in Co-rich amorphous wires. The study, which was conducted in the context of the development of a current clamp based on GMI, considered torsion as a parameter of the influence of this sensor. Both diagonal, Z_11_, and off-diagonal, Z_21_, components of the impedance tensor were investigated. The samples were Co-rich wires with a 100 µ diameter. The wires were twisted positive and negative angles with respect to a reference position. For each component of the impedance, the intrinsic sensitivity and offset were measured as a function of the rotation angle. The results showed that the sensitivity of the diagonal component at a given working point slightly increased for angles between −90° to +90°, whereas the sensitivity was almost constant for the off-diagonal component at zero-field. The intrinsic offset in the diagonal configuration was almost unchanged for the rotation angles considered, whereas this offset increased in the off-diagonal configuration. Furthermore, the GMI ratio of Z_11_ was also measured as a function of the rotation angle for comparison purposes with known data. The maximum of this ratio was obtained for a rotation angle of about 50°.

## 1. Introduction

Giant Magneto-Impedance (GMI) is a significant change of the impedance of some soft magnetic materials when they are subjected to an external magnetic field. This change of the impedance is directly related to the change of the skin depth of the high-frequency current in the magnetic conductor through the change of the magnetic permeability of the material with the applied magnetic field [1].

The GMI effect is investigated for the realization of magnetic sensors. These sensors are based on the impedance measurement of the sensitive element. They combine excellent features such as high sensitivity and a large bandwidth (from DC to several megahertz). Despite its potential, this technology is in fact still not very mature, especially for industrial applications. To our knowledge, only a few “concrete” realizations or commercial versions of these sensors have been achieved [2,3,4,5,6,7]. The systematic use of GMI sensors requires a clear identification of the areas of application for which these sensors can have clear advantages when compared to other mature magnetic sensor technologies. Contactless electrical current measurement could be one of these areas. For such applications, the ability of the GMI sensor to measure both DC and AC magnetic fields with the same sensitive element is clearly an important feature. Moreover, the mechanical flexibility of some GMI elements (like amorphous wires) is another key and very useful advantage since it enables the sensitive wire to be deformed in order to be aligned with the magnetic field produced by the measured current.

The work presented in this paper, which deals with the torsion stress effect, was actually conducted in the context of a specific application. This concerns the use of GMI to realize a toroidal current clamp (probe), which is mechanically flexible (not rigid) and which allows for both DC and AC measurements with the same sensitive element. By far, satisfying these features could not be easily achieved using the magnetic sensor technologies available. In this context, GMI clearly has a decisive advantage.

The basic principle of the current clamp using GMI is quite simple. The GMI wire circles the conductor that carries the measured current *I_m_*. This current produces a circumferential field, *H_m_*, which is measured by the GMI element. In practice, the clamp must be opened to circle the conductor and then closed to perform the measurement. Such a use of the clamp involves repetitive mechanical stresses. The effect of these stresses, as parameters of influence, on the GMI response needs to be carefully investigated. Repetitive bending is one such type of mechanical stress. Torsion stress could also be involved. It actually depends on how the user manipulates the sensor to perform the measurement. In practice, both bending and torsion stresses could be combined, resulting in a global change of the GMI response. However, for obvious reasons of simplicity, the two effects were studied separately.

Unlike the tensile effect, which has been intensively investigated (References [8,9,10,11,12,13,14,15,16] provide a non-exhaustive list of these studies), the effect of bending and torsion stresses on GMI have been investigated far less in amorphous GMI wires. The bending stress effect on the GMI response in amorphous wires has been addressed in our recent work [17,18] and more recently in Reference [19].

To our knowledge, the torsion stress effect on diagonal GMI was studied in some publications [16,20,21,22,23,24,25,26]. These publications evidently have great merit. However, on the one hand, most of them deal mainly with the diagonal component of the impedance tensor. Moreover, only the change of the GMI ratio of this component was considered under torsion stress. While this ratio is frequently used as a factor of merit to quantify the GMI effect, it is not the most relevant quantity to take into account for the sensor implementation. In fact, some GMI sensitive elements could have a large GMI ratio and at the same time exhibit a low sensitivity around the working point [27]. In this paper, the change of the diagonal component under torsion stress was addressed with particular attention paid to the change of the most relevant quantities, namely the intrinsic offset and sensitivity at a given working point. Nevertheless, the GMI ratio was also briefly considered in this study for comparison purposes with other previous studies dealing with the torsion stress effect. On the other hand, to our knowledge, the impact of torsion on the off-diagonal component has only been studied in a few publications [28,29,30], despite the promising characteristics of this component for GMI sensors. The change in sensitivity and offset near the zero-field point was not investigated. This is why an investigation of the torsion stress effect on this off-diagonal component was conducted with a primary focus on the change of the intrinsic sensitivity and offset near the zero-field. In this study, the torsion stress was considered as a parameter of influence that affects the response of the GMI current sensor.

Section 2 of the paper presents, firstly, a brief overview of the quantities considered and the general approach of the study. Secondly, a description of the setup and the experimental conditions is given. The first results obtained are illustrated and discussed in Section 3.

## 2. Quantities Considered and Experimental Setup

**Figure 1:**
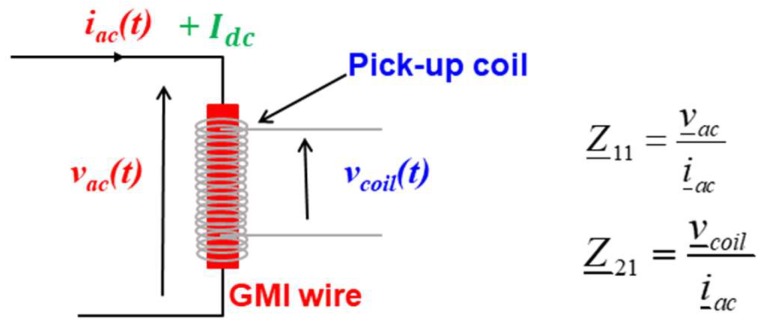
Principle of the diagonal and off-diagonal GMI sensor.

In a GMI sensor, the sensitive wire is supplied by a high-frequency current, *i_ac_*, of constant amplitude, as illustrated in Figure 1.

**Figure 2:**
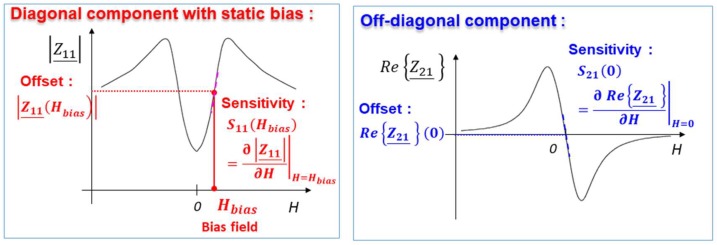
Typical behaviors of the diagonal and off-diagonal components of the impedance tensor in amorphous wires.

The impedance is a tensor which has two main components: the diagonal, ${\underline{Z}}_{11}$, and off-diagonal, ${\underline{Z}}_{21}$, components [31,32,33,34]. The typical characteristics of these components as functions of the magnetic field, *H*, are illustrated in Figure 2.

Since the behavior of the modulus of ${\underline{Z}}_{11}$ is nonlinear, a GMI sensor based on this component requires the use of a bias field (generally static bias), *H_bias_*, to fix a working point in the almost linear region. It is an axial external magnetic field, which could be produced using a coil or a permanent magnet, for example. This external magnetic bias gives rise to an offset which is related to the value of the impedance, *|Z*_11_(*H_bias_*)|, at this field. When the measured field, *H_m_*, is applied, the voltage, *v_ac_*, across the sensitive element is amplitude modulated by this field. After amplitude demodulation, and offset suppression, the output voltage of the sensor is obtained. This output is proportional to ${\frac{\partial \underline{Z_{11}}}{\partial H}|}_{H=H_{bias}}\times I_{ac}\times H_{m}$, where *I_ac_* is the amplitude of the excitation current. The intrinsic sensitivity, denoted as *S*_Ω −11,_ is defined as being derivative of the impedance curve at the bias point ($S_{\Omega -11}(H_{bias})={\frac{\partial \underline{\left|Z_{11}\right|}}{\partial H}|}_{H=H_{bias}}$).

It is also possible to introduce the voltage sensitivity, denoted as *S_v_*-_11_, which takes into account the amplitude, *I*_ac_, of the excitation current. This voltage sensitivity is defined by Equation (1)

(1) $$S_{v-11}(H_{bias})=S_{\Omega -11}(H_{bias})\times I_{ac}={\frac{\partial \underline{\left|Z_{11}\right|}}{\partial H}|}_{H=H_{bias}}\times I_{ac}$$

One or the other of these quantities will be used, indifferently, in this paper.

A GMI sensor based on the off-diagonal component requires, a priori, no external axial magnetic bias since both the real, Re{Z_21}, and the imaginary, Im{Z_21}, parts are intrinsically almost linear and asymmetric (odd symmetry) with respect to the zero-field point, as seen in Figure 2. However, the appearance of the off-diagonal component in wires with circumferential anisotropy requires the use of a DC current flowing in the wire and producing a circumferential field, as is mentioned in Section 3.2.

Without the loss of generality, and for simplicity reasons, only the real part, Re{Z_21}, is considered (the imaginary part exhibits similar behavior). The working point is around the zero- field. In this case, the offset is defined as the value of the real part at the zero-field, Re{Z_21}(0), which is almost zero. The intrinsic sensitivity, denoted as *S*_Ω*−*21_, is defined by the derivative of the curve at this same point (SΩ−21(0)=∂Re{Z_21}∂H|H=0).

In a similar way as for the diagonal component, the voltage sensitivity, denoted as *S*_*v*−21_, is defined by Equation (2)

(2) Sv−21(0)=SΩ−21(0) . Iac=∂Re{Z_21}∂H|H=0 Iac

For the intended application, which is the development of a GMI current clamp, the GMI sensor should be implemented in closed-loop. The intrinsic voltage sensitivity, combined with the gain of amplification of the conditioning electronics, determines the open-loop gain. This open-loop gain has to be “high enough” to guarantee a well-regulated closed-loop. In this way, the sensor output is less dependent on the imperfections of the open-loop. Some advantages of the closed-operation include improved linearity, temperature dependence, and hysteresis of the sensor response as well as a higher dynamic range [35]. The change of the intrinsic sensitivity under quantities of influence like torsion stress must be known in order to compensate for it, if necessary. The general goal is to maintain a “high enough” open-loop gain.

That is why the investigation of the change of the intrinsic sensitivity is investigated under torsion for both diagonal and off-diagonal components. In addition, the change of the offset, which is another practical issue, is also considered. Finally, for comparison purposes with other published works only, the evolution of the GMI ratio for the diagonal component, ${\underline{Z}}_{11}$, under torsion is recalled.

**Figure 3:**
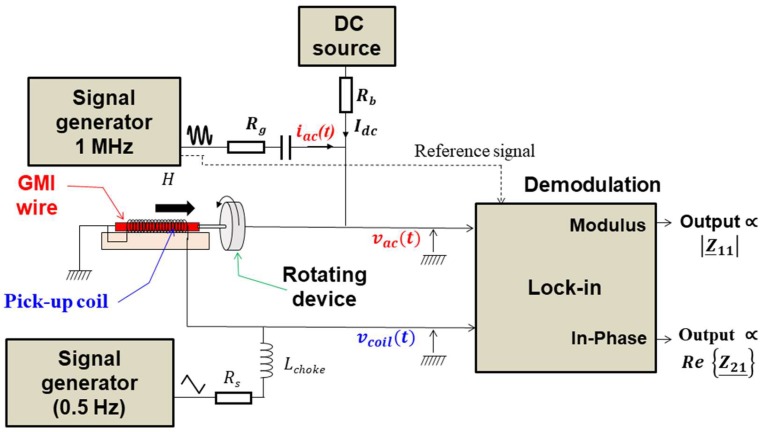
Schematic of the experimental setup for the investigation of the torsion stress effect on GMI in amorphous wires.

A schematic of the experimental setup is shown in Figure 3.

The samples studied were Co-rich amorphous wires (Co-Fe-Si-B) with a 100 µm diameter from Unitika. These wires exhibit nearly zero magnetostriction. A pick-up coil was wound around each wire. The GMI element was soldered to a rotation device. The twisting of the wire was performed by rotating this device, which ensures the application of a torsion with a defined rotation angle. Indeed, this rotation device was graduated to measure the rotation angle starting from a reference position corresponding to the zero angle.

The wire was supplied by the high-frequency current, *i_ac_*, using a signal generator and a voltage-to-current convertor made by the resistor *Rg*. In the same way, a DC current could be supplied to the wire using a DC source and the resistor *R_b_.* The voltages, *v_ac_*, across the wire, and *v_coil_*, across the pick-up coil, were demodulated using a lock-in amplifier. The output voltages of this lock-in amplifier are proportional to the diagonal and off-diagonal components. A low-frequency (0.2 Hz) sweeping of the magnetic field was applied to the sensitive element using the same pick-up coil. An electrical “separation” between this low-frequency section and the high-frequency one was made using a choke inductor *L_choke_.*

## 3. Results and Discussion

### 3.1. The GMI Ratio of the Diagonal Component

**Figure 4:**
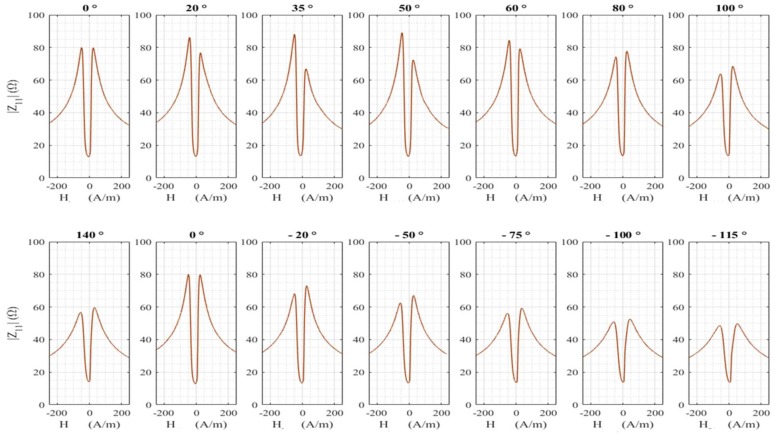
Modulus of the diagonal component for different rotation angles of the sensitive element.

For this experiment, the GMI wire used was 6.5 cm long with a pick-up coil of about 1000 turns. The high-frequency current, *i_ac_*, had a frequency of 1 MHz and an amplitude of 3 mA. No DC current was supplied to the wire. Figure 4 shows the modulus of the diagonal component for positive and negative rotation angles of the sensitive element. The zero-degree angle corresponded to the position at which the wire was initially soldered.

It can be seen that there is obviously a net change of the impedance curve with the torsion. This change can be quantified using the GMI ratio defined by Equation (3)

(3) $$\Delta \left|Z_{11}\right|/\left|Z_{11}\right|\left(\%\right)=\frac{\left|\left|Z_{11}\right|\left(H\right)-\left|Z_{11}\right|\left(H_{\max}\right)\right|}{\begin{array}{c} \left|Z_{11}\right|\left(H_{\max}\right) \end{array}}\times 100$$

where $H_{\max}$ is the maximum available field.

**Figure 5:**
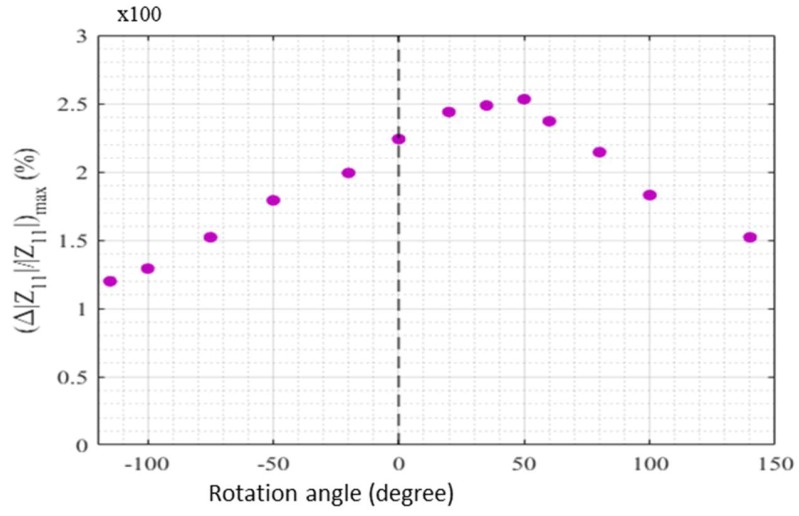
Maximum of the GMI ratio as a function of the rotation angle.

For each rotation angle, this ratio has a maximum noted ${\left(\Delta \left|Z_{11}\right|/\left|Z_{11}\right|\right)}_{\max}$. This maximum is plotted in Figure 5 as a function of the rotation angle. It can be seen that the behavior is not symmetrical for positive and negative rotation angles. The maximum is obtained for a rotation angle near 50° and not for a zero degree.

These results are very close to the ones reported in Ref. [16], where a similar behavior in as-cast Co-rich amorphous wires has been observed. The change in the GMI response was interpreted based on a change in the domain structure. Indeed, in the case of negative magnetostriction, the domain structure in the GMI wire consists of a core, which is axially magnetized, and an outer shell with circular magnetization [36,37,38,39,40]. The wires used in our experiments have a nearly zero magnetostriction constant. In this case, it is usually assumed that the magnetic structure is roughly the same as the structure encountered in negative magnetostriction wires [16,24,36,37,38]. The torsion modifies this spatial distribution of the magnetization close to the surface of the wire. This results in a modification of the circumferential permeability and consequently of the GMI response with torsion. The asymmetry in the GMI ratio in Figure 5 may indicate that there were already preexisting torsional stresses in the wire, which may be due to the fabrication process. Some of these preexisting torsional stresses have been partially compensated for a torsion corresponding to a rotation angle of 50° [16,23]. In this context, it is to be noted that the torsion applied could in fact not completely compensate for these internal stresses due to the complexity of their distribution [23].

### 3.2. Offset and Sensitivity of the Diagonal and Off-Diagonal Components

For this experiment, the GMI wire used was 9 cm long with a pick-up coil of about 1400 turns. A high-frequency current, *i_ac_*, of 1 MHz/5 mA was used. A DC current, *I_dc_* = *5* mA, was also supplied to the wire. This current is required for the significant appearance of the off-diagonal component in wires with circumferential anisotropy [31,32,33,34]. In addition, this current may make it possible to obtain an anhysteretic field dependence of the impedance when its value exceeds a certain threshold [26,28,29,30].

It is worth noting that the use of a relatively low frequency of 1 MHz is justified by practical considerations related to the parasitic resonance of the pick-up coil. This resonance is due to the LC circuit formed by the inductance of the coil and the stray capacitance between the turns of the winding. Indeed, for the off-diagonal configuration, the voltage sensitivity increases up to a maximum, which is obtained for an optimum frequency. This sensitivity then decreases when the frequency increases [29,41]. The optimum frequency is lower than the frequency of parasitic resonance of the pick-up coil. For the GMI sensitive element and pick-up coil used, this optimum was experimentally found to be about 1 MHz. Furthermore, the amplitude of the high-frequency current was chosen to be relatively low to avoid the nonlinear regime of the GMI and to thereby guarantee a linear relationship between the voltage and the current in the wire [42]. Finally, for a given AC current, the value (5 mA) of the DC current was used as a trade-off between the sensitivity and the reduction of the level of low-frequency intrinsic magnetic noise of the sensitive element [43].

In summary, in practice, the set of values for the excitation (frequency, amplitude of the AC current, and value of the DC current) was chosen to optimize the sensitivity and noise (under zero torsion stress) while avoiding both nonlinear effects of the GMI and the paratactic resonance of the pick-up coil. The impact of the torsion stress, as an influence parameter, was studied under these real conditions of implementation of the GMI current clamp.

**Figure 6:**
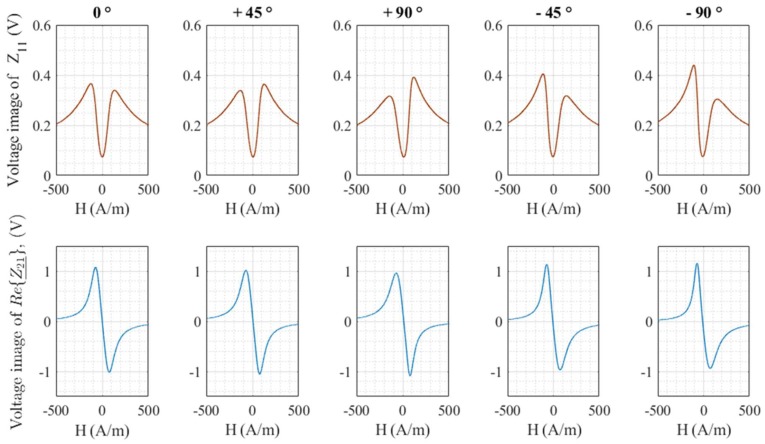
Voltages proportional to the diagonal and off-diagonal components as a function of the magnetic field for different rotation angles.

Figure 6 shows voltages proportional to the modulus of the diagonal component and to the real part, Re{Z_21}, of the off-diagonal component for a few rotation angles.

A general change of both components with torsion is observed. The torsion seems to induce an asymmetry of the curve for the diagonal component, whereas the odd symmetry of the off-diagonal component seems to be well-preserved.

**Figure 7:**
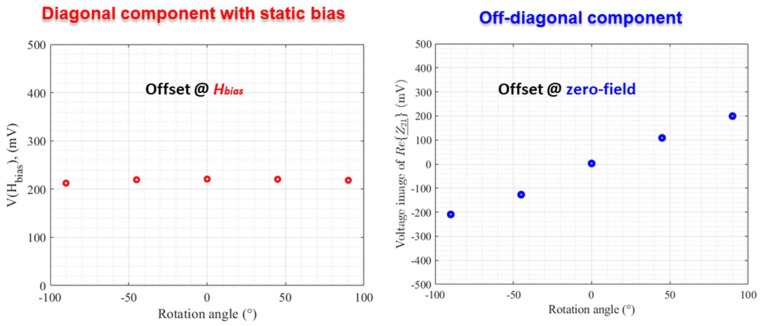
Change of the offset of the diagonal and off-diagonal components as a function of the rotation angle.

Voltages proportional to the offsets of these components are plotted in Figure 7.

For the diagonal component, only a small change of this offset, at a given bias, was observed for the range of rotation angles considered. In the off-diagonal case, the offset near the zero-field point exhibited a significant change. At this first stage of investigation, and in a first approximation, the change of the offset of this component at the zero-field could be roughly interpreted by knowing the origin of the voltage induced in the pick-up coil. Indeed, in the absence of torsion stress, this induced voltage results from the appearance of an AC longitudinal magnetization in the GMI wire, which is circumferentially magnetized by the excitation current, *i_ac_*. To observe this longitudinal component of the magnetization, the static magnetization must follow a helical path around the direction of the excitation current [31,32,33,34]. In the case of a GMI wire with an almost circumferential anisotropy, as is the case with the wire used in this study, a DC static current, *I_dc_*, superimposed to the excitation current, *i_ac_*, is required. The static circumferential field produced by *I_dc_*, combined with the longitudinal external measured field, allows the magnetization to have a helical path. Hence, in the absence of an external measured field, there is no longitudinal magnetization and the coil voltage is almost null. In other words, the offset is roughly null at the zero-field. When the wire is twisted, a helical magnetic anisotropy could be induced so that an AC longitudinal magnetization component appears. This magnetization component gives rise to a coil voltage, even when the external magnetic field is not applied, that is to say, the offset at the zero-field is not null.

**Figure 8:**
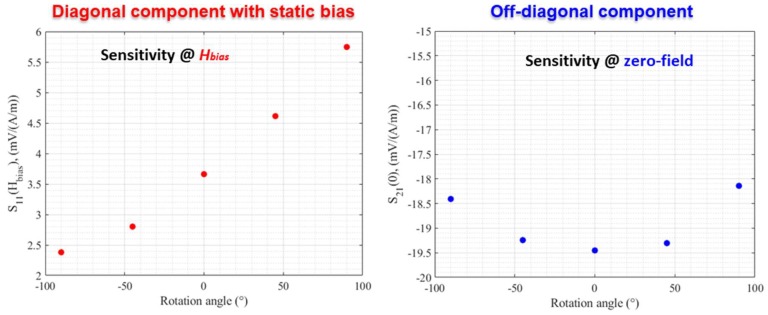
Change in the sensitivity of the diagonal and off-diagonal components as a function of the rotation angle.

For the sensitivity of these components, the tendency of change was the opposite, as illustrated in Figure 8. This sensitivity was almost unchanged for the off-diagonal component near the zero-field point (a maximum change from −19.5 mV/(A/m) at zero angle to about −18 mV/(A/m) at 90 °). In the case of the diagonal component, a significant change in the sensitivity at the bias field was observed.

For this diagonal component, a rough estimation of the intrinsic sensitivity could generally be given by the ratio between the field of the maximum of the impedance (which is closely related to the field of anisotropy) and the value of the impedance at this field [27]. For the data in Figure 6, this ratio increased, under torsion stress, in some linear behavior, from 0.42 Ω/(A/m) at −90° to 0.6 Ω/(A/m) at 90°. At least, this general trend is consistent with the almost linear increase in the sensitivity measured in Figure 8 in the range of rotation angles considered.

The experimental results showed a better offset stability for the diagonal component under torsion. The sensitivity change was less important for the off-diagonal component near the zero-field point.

The torsion, as a parameter of influence of the GMI current sensor, directly influences the choice of one or the other of the two components for sensor implementation. Usually, the offset change is an issue, especially when the sensor in intended to measure DC magnetic fields. In this case, the use of the diagonal component may be preferred. However, in this case, attention should be paid to the gain of the open-loop, which changes with the torsion stress, through the change in the intrinsic sensitivity. The off-diagonal component still exhibits the advantage of odd symmetry, which was preserved under torsion stress for the set of excitation parameters used. This preserved odd symmetry near the zero-field point is important since it still allows for the implementation of the off-diagonal sensor without making use of an axial bias field to fix the working point. This greatly simplifies the design of the sensor.

Finally, it should be noted that the results presented were obtained for one set of excitation parameters that allowed maximum sensitivity under zero torsion stress. In practice, this is a pragmatic approach for considering the problem. However, both diagonal and off-diagonal components depend on the high-frequency excitation current and on the DC current.
